# Theory of change for addressing sex and gender bias, invisibility and exclusion in Australian health and medical research, policy and practice

**DOI:** 10.1186/s12961-024-01173-z

**Published:** 2024-07-15

**Authors:** Thomas Gadsden, Laura Hallam, Cheryl Carcel, Robyn Norton, Mark Woodward, Louise Chappell, Laura E. Downey

**Affiliations:** 1 The George Institute for Global Health, University of New South Wales, Sydney, Australia; 2 Australian Human Rights Institute, University of New South Wales, Sydney, Australia; 3 The George Institute for Global Health, Imperial College London, London, United Kingdom; 4 The Human Rights Institute, University of New South Wales, Sydney, Australia

**Keywords:** Theory of change, Sex and gender, Medical research, Health policy, Evidence-based practice

## Abstract

Sex and gender are inadequately considered in health and medical research, policy and practice, leading to preventable disparities in health and wellbeing. Several global institutions, journals, and funding bodies have developed policies and guidelines to improve the inclusion of diverse participants and consideration of sex and gender in research design and reporting and the delivery of clinical care. However, according to recent evaluations, these policies have had limited impact on the inclusion of diverse research participants, adequate reporting of sex and gender data and reducing preventable inequities in access to, and quality provision of, healthcare. In Australia, the Sex and Gender Policies in Medical Research (SGPMR) project aims to address sex and gender bias in health and medical research by (i) examining how sex and gender are currently considered in Australian research policy and practice; (ii) working with stakeholders to develop policy interventions; and (iii) understanding the wider impacts, including economic, of improved sex and gender consideration in Australian health and medical research. In this paper we describe the development of a theory of change (ToC) for the SGPMR project. The ToC evolved from a two-stage process consisting of key stakeholder interviews and a consultation event. The ToC aims to identify the pathways to impact from improved consideration of sex and gender in health and medical research, policy and practice, and highlight how key activities and policy levers can lead to improvements in clinical practice and health outcomes. In describing the development of the ToC, we present an entirely novel framework for outlining how sex and gender can be appropriately considered within the confines of health and medical research, policy and practice.

**Supplementary Information:**

The online version contains supplementary material available at 10.1186/s12961-024-01173-z.

## Contributions to the literature

- Inadequate consideration of sex and gender in health and medical research, policy and practice contributes to inequity in the distribution of health.
- No framework for guiding the consideration of sex and gender in health and medical research, policy and practice currently exists.
- The theory of change presented in this paper presents an entirely novel and important theoretical scaffold for institutions and organizations to use when considering how to actively enhance sex and gender awareness and inclusivity in their activity.
- Reorienting practice through informed action can contribute to improving population health and economic wellbeing by enhancing sex and gender equity.

## Introduction

Sex and gender are important determinants of health but are often inadequately considered in health and medical research, policy and practice [1–3]. Documented issues include exclusion or underrepresentation of cis women, trans women and men, those with intersex characteristics and non-binary populations in research participation and lack of sex and gender disaggregation and interpretation of data (see Table 1 for definitions of key concepts) [2, 4]. As health and medical research forms the evidence base that many clinical guidelines and health policies are based on, this can lead to disparities in diagnosis, treatment and health outcomes. Sex- and gender-based health disparities have been identified in many conditions, such as cardiovascular disease [5, 6], stroke [7] and pain [8], and heath areas such as screening, diagnosis [9] and reactions to pharmacological treatments [10].

**Table 1:** Definitions of key terminology (source: [23])

Term	Definition
Equity	Equity is concerned with everyone achieving equal outcomes, recognizing that each individual has a different set of circumstances and starting point that requires a tailored approach to support this achievement
Gender	Refers to the way in which a person identifies or expresses themselves, including behaviour, attitudes, appearance and habits. A person’s gender identity or gender expression is not always binary (man or woman; see non-binary) and may change over time. Gender is distinct from sex. Gender attitudes and behaviours are complex and change across time and place, and gender should be understood in relation to other social categories (see intersectionality). Gender also encompasses gender norms and gender relations
Intersectionality	An analytical framework for understanding how multiple social and political identities (including but not limited to gender, caste, sex, race, ethnicity, sexuality, religion, disability, physical appearance, etc.) may produce different forms of advantages and disadvantages for individuals across social systems, including in health and medical research
Sex	Sex for humans is a legal status, classified as either male or female in most jurisdictions, and which is typically presumed or observed at birth on the basis of external sex characteristics. While typically based on these sex characteristics, a person’s sex can change over the course of their lifetime and may differ from their sex recorded at birth

In response to these health disparities, in recent decades several research institutions have developed policies and guidelines to improve the inclusion of diverse participants and consideration of sex and gender in research design and reporting [11–15]. New and updated policies increasingly highlight broader issues of equity and intersectionality alongside sex and gender considerations [16]. The aims and potential impacts of these policies include improved rigour, ethics and reproducibility of science, counteracting existing biases and exclusionary practices, understanding health differences and inequities, informing and improving health policy and care and advancing gender equality, diversity and inclusion. Positive initiatives towards updating university science and medical curricula [17–19], clinical protocols and guidelines [6, 20] and public and global health programs to explicitly incorporate sex and gender considerations have also been reported [21, 22].

Evaluations of these policies and guidelines report mixed results on health and medical research design and reporting to date [24–26]. For instance, an evaluation of the Canadian Sex- and Gender-Based Analysis policy found increased explicit consideration of sex and gender in grant applications [27], while other policy evaluations have found limited impact on the inclusion of women and marginalized community members in study populations and on the disaggregation and analysis of results by sex and gender [28–30]. A range of barriers to progress have also been reported, including limited awareness, a lack of training and resources among personnel across the medical research pipeline, and the absence of adequate accountability and monitoring mechanisms by regulators, including government agencies, funders and universities, among others [31–33].

In Australia, the Sex and Gender Policies in Medical Research (SGPMR) project was established to understand the current state of play in relation to the explicit consideration of sex and gender in medical research and practice [34]. SGPMR is a philanthropically funded initiative with three primary aims: (i) to understand whether and how sex and gender are addressed in current research policy and practice in Australia; (ii) to work with stakeholders to co-develop policy interventions; and (iii) to understand the wider impacts, including economic impacts, of improving sex and gender consideration in Australian health and medical research [35]. Work to date has demonstrated that sex and gender are under-reported in research articles published in Australia’s top ten medical journals in 2020 [3] and that the content of academic journals dedicated to women’s health remains largely focussed on reproductive health topics, with few articles targeting the major causes of morbidity and mortality in women [36].

Common to each of these disparate initiatives is an implied shared understanding in the notion that explicit consideration of sex and gender in research design, policy and operations leads to better data and evidence-based practice, which in turn leads to better health outcomes. As has been previously argued [37], a clearly articulated explicit framework outlining the causal pathways by which better data and gender-sensitive practice would lead to better health would ensure that the opportunity for misunderstanding is minimized and enhance the coordination of efforts to achieve common goals and the ability to create and evaluate their impact enhanced. A Theory of Change (ToC), defined as “an explicit process of thinking through and documenting how a program or intervention is supposed to work, why it will work, who it will benefit and the conditions required for success”, is an increasingly common means in which to articulate a shared vision and map logical pathways to impact towards addressing a problem or issue [38]. This methodology is increasingly used in public health and evaluation frameworks to articulate how an intervention can achieve long-term impact by identifying and depicting causal pathways from activities to outputs and outcomes and the key mechanisms, barriers, and facilitators underpinning these causal pathways [39–42]. Although ToCs are typically developed for discrete projects or interventions, they have been used to promote shared ownership and understanding among stakeholders for broader initiatives, such as strengthening sector-wide response to human immunodeficiency virus (HIV) in Papua New Guinea [43] and multi-sector urban planning initiatives [44].

In this paper, we outline the development of a ToC to identify the pathways through which improved consideration of sex and gender in health and medical research, policy and practice could impact social and economic health outcomes. The objectives of this work are twofold: (1) to fill an important knowledge-implementation gap in the literature by explicitly documenting the problem, the desired impact and how engaging in certain activities can contribute towards achievement of positive change across the evidence, policy and practice pipeline; and (2) to situate the activities of the aforementioned SGPMR project in this wider context to guide future project activities aimed at creating impact in the Australian health and medical research sector.

## Methods

### Study design

This study followed best practice guidelines for developing a ToC, involving a wide range of stakeholders and end-users, ensuring rigorous evidence-based discussions through participatory research methods and engaging in an iterative process of refinement [45, 46]. These recommendations are reflected by the iterative six-step process followed in this study: (1) initial mapping of key concepts and considerations; (2) stakeholder interviews; (3) draft ToC development; (4) stakeholder consultation workshop; (5) revised ToC development; and (6) stakeholder review. Table 2 outlines the goals and methods of each of the steps. For transparency, this study is reported against the Standards for Reporting Qualitative Research (Supplementary file 1) and the Checklist for reporting ToC in Public Health Interventions (Supplementary file 2) [45].

**Table 2:** Outline of the six steps used to develop the theory of change map

Step	Task	Method
1: Initial mapping	Development of a structural framework to guide ToC development	Review of ToC literature, review of sex and gender research policy literature, mapping of SGPMR project activities including planned outputs and goals
2: Stakeholder interviews	Consultation with project stakeholders to inform a draft ToC	Semi-structured interviews with project stakeholders and thematic analysis of interview responses
3: Draft ToC development	Creation of a draft of the ToC using outputs from step 1 and 2	Development of a draft ToC based on the thematic analysis of the interview data
4: Stakeholder workshop	Refinement of the draft ToC with stakeholder input	Online stakeholder consultation workshop where draft ToC was presented for feedback through a facilitated discussion
5: ToC revision	Development of a second, graphically designed draft of the ToC	Integration of input from stakeholder workshop into ToC. Engagement with graphic designer to execute final design
6: Stakeholder revision	Receipt of final feedback on the second draft ToC from project stakeholders	Online peer review of second draft ToC by project stakeholders via email. Feedback was collated, synthesized and integrated into the final ToC

### Data collection

#### Initial mapping

A horizon scanning exercise was undertaken to identify literature of importance to this project by using PubMed and Google Scholar searches with different combinations of the key words “sex”, “gender”, “health” “medical” “research” “policy” “framework” “logic” and “theory of change”. Titles and abstracts were screened and full papers reviewed for literature identified as potentially relevant. No evidence was identified that directly addressed the development of a framework for explicit consideration of sex and gender in medical research, policy and practice. Literature on the development and use of a ToC was reviewed to determine the most appropriate structural framework for the purposes of this ToC. The approach chosen draws upon programmatic theory described as “deal[ing] with the mechanisms that intervene between the delivery of a program service and the occurrence of outcomes of interest” [29]. This approach requires that all activities and their intended outputs, outcomes and impacts are identified and then mapped to a ToC structural framework. Table 3 defines each component of the ToC structural framework used in this exercise. Barriers and facilitators to reaching the intended outcomes and impacts were also considered.

**Table 3:** Elements of the theory of change structural framework

Element	Definition
Problems	The issue(s) that the program of work aims to address
Activities	Actions or interventions needed to address the problems
Outputs	Products of the activities
Outcomes	Envisaged changed or developments during or after the program of work
Impacts	Long-term goals of the program of work

#### Study participants

Participation in the study was restricted to members of the SGPMR project [34]. There are 24 SGPMR project members in total: 8 principal investigators (PI) and an advisory group of 16 members. Members represent government, cisgender, trans, intersex, non-binary and indigenous community groups, as well as multidisciplinary academics with expertise in health and medicine, gender, human rights, policy, clinical care, regulation and community engagement.

#### Semi-structured interviews

All 24 individuals associated with the SGPMR project were invited via email to participate in a semi-structured interview. Interviews were conducted by one of three members of the core ToC research team (L.D., L.H., T.G.) who all have experience in qualitative research. Semi-structured interviews of 40–60 min were conducted, either online, via Zoom or in person. The predetermined interview guide included a brief introduction to ToC methodology and questions were organized around the structural framework to obtain views on the key problems, activities, outputs, outcomes and impacts that related to the SGPMR project and sex and gender in the health and medical research sector (Supplementary file 3). Questions were primarily framed in relation to the SGPMR project, thereby concerning the Australian context.

Interviews were audio recorded, with the written consent of participants. The research team reviewed the audio transcripts produced by the Zoom transcribe function alongside the audio recordings to develop an accurate transcript for each interview. Interview transcripts were coded using NVivo 12. Interview responses were mapped deductively to the elements of the structural framework and analysed inductively to identify common themes across interviews. Codes were discussed between the core ToC research team iteratively and the final list of codes was used to develop the draft ToC. Codes addressing similar themes were combined where possible.

#### Consultation workshop

All 24 members of the SGPMR project, regardless of whether they participated in an interview, were invited to provide further input on the draft ToC by participating in a 2-h online workshop on 23 September 2022. The draft ToC was presented by the lead facilitator (L.D.) and each element was presented for discussion and feedback amongst the group. Content, language and structure of the draft were all reviewed, with further explanation by the facilitators and input from stakeholders. The discussion was audio-recorded with the consent of participants and one facilitator took extensive notes (T.G.), which were used by the research team to make amendments to the draft.

#### Ethics

This study received ethical approval from the Human Research Ethical Approval Panel (HREAP) at the University of New South Wales (HC220443). All participants provided written consent to participate in this study.

## Results

### Participation

A total of 15 individuals (4 PI members, 11 advisory group members), participated in semi-structured interviews, and 7 individuals (4 PI members, 3 advisory group members) participated in the 2-h online workshop. Participants represented expertise in clinical research, social sciences, academic, non-government community-based organizations and government. The most common reason for declining participation was unavailability. A total of 12 individuals (5 PI members and 7 advisory group members) provided peer review of the draft ToC schematic. In total, 19 out of 24 (79.2%) individuals associated with the SGPMR project provided some input into the ToC.

### Theory of change

**Fig. 1:**
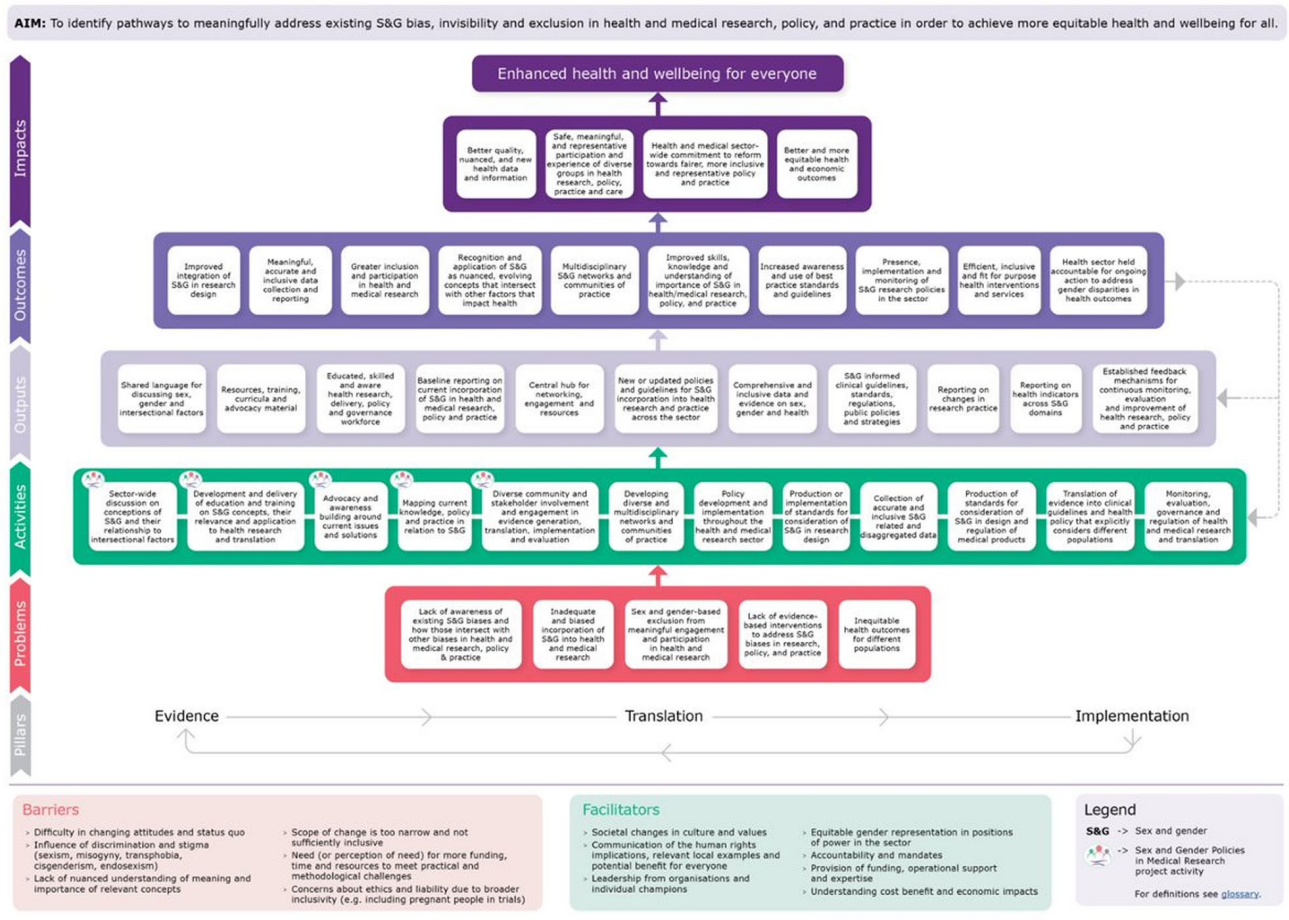
Theory of change for addressing sex and gender bias, invisibility and exclusion in health and medical research, policy and practice

The ToC is shown in Fig. 1 (see Supplementary File 4 for a tabulated version). Results are structured according to the elements of the ToC (problem statement, required activities, outputs and outcomes, impact). Boxes in the ToC are referred to in the results below numbered from left to right across the diagram for each element of the framework.

#### Problems

Five key problems regarding the current consideration of sex and gender in health and medical research, policy and practice were identified for inclusion in the ToC: (1) lack of awareness of existing sex and gender biases and how those intersect with other biases in health and medical research, policy and practice; (2) Inadequate and biased incorporation of sex and gender into health and medical research; (3) sex and gender-based exclusion from meaningful engagement and participation in health and medical research; (4) lack of evidence-based interventions to address sex and gender biases in research, policy and practice; and (5) inequitable health outcomes between different populations.

Interviewees identified various forms of bias in research against different groups on the basis of sex and gender, which lead to science that is not rigorous or representative. Several potential reasons were proposed for inadequate consideration of sex and gender in research including poor societal understanding of sex and gender, particularly the predominance of a binary concept of sex and gender, inadequate data collection, lack of inclusion and analysis by sex and gender, too strong a focus on biological sex and a lack of consideration of intersectional factors, including race, social, economic and other factors. Other issues included the exclusion of marginalized communities from participating in research, a lack of adequate community consultation and input into research projects, which can lead to the design and funding of unethical research. Stakeholders discussed inequitable access to healthcare, which contributes to poorer health outcomes, particularly for women, transgender and gender-diverse people and people with intersex variations, and associated economic losses.

#### Activities

A total of 12 activities with potential to address the problems raised above emerged from participants’ responses. As a key starting point, respondents emphasized the need for improved understanding of the terms sex and gender, both within the health and medical research sector and societally. Respondents stressed that the complexity of these concepts and their evolving nature required a suitable conceptual framework that could be used to guide other activities. Accounting for intersectionality was also highlighted in the workshop as a vital component for any such framework to consider.

These themes are reflected in the ToC through the inclusion of activities that relate to building knowledge and awareness, education, training and advocacy:

1. Sector-wide discussion on conceptions of sex and gender and their relationship to intersectional factors;
2. Development and delivery of education and training on sex and gender concepts, their relevance and application to health research and translation;
3. Advocacy and awareness building around current issues and solutions;
4. Mapping current knowledge, policy and practice in relation to sex and gender.
  Two activities focussed on the need for meaningful consultation and the building of networks and partnerships to share knowledge and expertise and facilitate change that appropriately accounts for the needs of diverse communities:
5. Diverse community and stakeholder involvement and engagement in evidence generation, translation, implementation and evaluation;
6. Developing diverse and multidisciplinary networks and communities of practice.
  Several activities focussed on changing research practice and the development of policies, guidelines and standards to assist this change:
7. Policy development and implementation throughout the health and medical research sector;
8. Production or implementation of standards for consideration of sex and gender in research design;
9. Collection of accurate and inclusive sex- and gender-related and disaggregated data. Many stakeholders suggested that the Australian Bureau of Statistics Standard for Sex, Gender, Variations of Sex Characteristics and Sexual Orientation Variables [47] is a locally relevant example of such a standard that can be further implemented and used to guide data collection.
  Expanding beyond health and medical research, two activities focussed on supporting translation of research evidence into practice:
10. Production of standards for consideration of sex and gender in design and regulation of medical products such as drugs and devices.
11. Translation of evidence into clinical guidelines and health policy that explicitly considers different populations.
  The last activity underpins the implementation pillar and applies to all previous activities:
12. Monitoring, evaluation, governance and regulation of health and medical research and translation.
  Participants repeatedly addressed the need for monitoring and evaluating the impact of interventions such as education and training, and changes to health and medical research, policy and practice. Participants emphasized the importance of a consistent process of review and adaptation over time, based on monitoring and evaluation, and taking account of the dynamic nature of sex- and gender-based research, concepts and terminology. It was also highlighted that this process should go beyond tokenistic metrics to understand and evaluate how institutional change occurs.

#### Outputs

A total of 11 outputs were identified as emerging from the activities. These were:

1. Shared language for discussing sex, gender and intersectional factors;
2. Resources, training, curricula and advocacy material;
3. Educated, skilled and aware health research, delivery, policy and governance workforce;
4. Baseline reporting on current incorporation of sex and gender in health and medical research, policy and practice;
5. Central hub for networking, engagement and resources.
6. New or updated policies and guidelines for sex and gender incorporation into health research and practice across the sector;
7. Comprehensive and inclusive data and evidence on sex, gender and health;
8. Sex- and gender-informed clinical guidelines, standards, regulations, public policies and strategies;
9. Reporting on changes in research practice;
10. Reporting on health indicators across sex and gender domains; and
11. Established feedback mechanisms for continuous monitoring, evaluation and improvement of health research, policy and practice.

#### Outcomes

Outcomes reflected the priority activities raised by participants. They strongly felt that the identified activities would lead to improvements in research practice, including (1) improved integration of sex and gender in research design; (2) meaningful, accurate and inclusive data collection and reporting; (3) greater inclusion and participation in health and medical research; and (4) presence, implementation and monitoring of sex and gender research policies in the sector.

Other outcomes reflected the impacts of training, educational and advocacy activities: (5) recognition and application of sex and gender as nuanced, evolving concepts that intersect with other factors that impact health; (6) multidisciplinary sex and gender networks and communities of practice; (7) improved skills, knowledge and understanding of importance of sex and gender in health/medical research, policy and practice; and (8) increased awareness and use of best practice standards and guidelines.

Respondents also identified outcomes that may result from the translation of policies into more appropriate healthcare services and treatment: (9) efficient, inclusive and fit for purpose health interventions and services. This encompassed a variety of possible outcomes raised by stakeholders, including clinicians being able to provide inclusive and appropriate care, better and more cost-effective healthcare delivery, more targeted support for particular populations and more robust medical products. Respondents also felt that the existence of adequate monitoring and evaluation processes would result in an outcome whereby the (10) health sector (is) held accountable for ongoing action to address gender disparities in health outcomes.

#### Impacts

Four impacts were identified which fed into the overarching impact of: enhanced health and wellbeing for everyone. Typically, sex and gender research is narrowly viewed as only relevant to women and other marginalized communities, yet the participants emphasized that identifying and evaluating health data benefits all population groups. This was reinforced by the four sub-impacts that were identified: (1) better-quality, nuanced and new health data and information; (2) safe, meaningful and representative participation and experience of diverse groups in health research, policy, practice and care; (3) health and medical sector-wide commitment to reform towards fairer, more inclusive and representative policy and practice; and (4) better and more equitable health and economic outcomes for all.

#### Barriers and facilitators

Key barriers and facilitators to change were also identified by respondents. These were applicable across the entire ToC map and not just to the achievement of specific outputs, outcomes or impacts.

Two barriers focussed on the influence of entrenched systems and beliefs, including difficulty in changing attitudes and status quo and the influence of discrimination and stigma. Practical barriers included the need or perception of need for more funding, time and resources to meet practical and methodological challenges. Another barrier to change was concern regarding ethics or liability when broadening research inclusivity, particularly when including those who are pregnant or lactating in clinical trials.

Facilitators included societal changes in culture and values that would increase receptivity to change. Another facilitator was leadership, with leadership from organizations and individual champions as well as the equitable gender representation in positions of power across the sector being facilitators for change. Organizations across the sector can also facilitate change through the provision of funding, operational support and expertise and the implementation of accountability measures and mandates.

## Discussion

To the best of our knowledge, this is the first theory of change (ToC) to explicitly outline a common understanding of the sex and gender bias, invisibility and exclusion in health and medical research, policy and practice and outline clear actions and pathways to impact towards enhanced health and wellbeing for all. This work therefore fills an important knowledge-implementation gap in the literature by demonstrating how changes in research policy and practice may create wider impact and the explicit assumptions underlying the guiding future activities and discussion in the field. We identify a range of required actions across evidence generation, translation and implementation that contextualizes the work that many in the sector are already doing, situates the activities of the SGPMR project in this wider context and has the potential to inform the development of future activities. Further, the overarching impact of enhanced health and wellbeing for all is a unifying goal for people working across these sectors, and thus this ToC can be used to reinforce the need to address sex and gender bias, invisibility and exclusion to achieve this impact.

In providing a scaffold for how positive change might occur across medical research, policy and practice through clearly articulated pathways to impact, this ToC also provides important theoretical underpinning to published estimates of macro-level return on investment in gender inclusive research and practice. For example, the donor Women’s Health Access Matters (WHAM) reported that investment of USD$ 300 million in women’s health research across three diseases could result in returns to the economy in excess of USD$ 13 billion by way of improvement in population health and economic productivity [48]. Assumptions made in the WHAM report regarding how increased investment in research leads to improved health are afforded a more nuanced understanding when considered alongside the pathways to impact articulated in this ToC.

Activities articulated by study participants and represented in the ToC align well with the limited literature that describes initiatives already underway that consider and address disparity in scientific and medical practice. For example, White et al. summarize lessons learnt from funding agencies in developing policies for sex and gender consideration in medical research and identify awareness building, education and collaboration between institutions and continual monitoring and evaluation as necessary to facilitate impact [25]. Initiatives such as Gendered Innovations and Global Health 50/50 are also actively engaged in building awareness of the need for and value in gender diverse participation in health and medical research and practice and provide guidance to different types of organizations to enhance their practice in this respect whilst monitoring progress against gender inclusion within the global health sector [21, 49].

For those who are already working on specific activities such as developing or updating policies and guidelines to impact research practice [16, 50, 51], this ToC can help contextualize this change, inform design and encourage organizations to consider what parallel activities might be needed, such as education and training, consultation with key stakeholders and clarification of concepts across the sector. The ToC demonstrates the importance to those working to address sex and gender issues in evidence, translation and implementation of the need to coordinate their efforts and ensure monitoring and evaluation is communicated to inform practice throughout the pipeline.

### Implications for the SGPMR project

The development of this ToC has various implications for the SGPMR project. First, while the ToC spans far beyond than the scope of the project, it supports project affiliates to identify activities to which they can contribute and situate their efforts in a wider change context. Further, as engaging with stakeholders across the health and medical research sector is an activity of the project, this ToC can be used as an advocacy tool to demonstrate the need for change and the role of different organizations in contributing to that change [34, 35].

Second, this work also served as a useful activity to reach consensus on the key issues to be addressed and the desired impact of the project. It also facilitated discussion regarding the limitations of this project in achieving long-term impact on health outcomes due to the concentration of activities at the evidence end of the pipeline. Further, this process highlighted the diverse perspectives and priorities of different project stakeholders, related to issues faced by certain populations (namely, cis women, transgender women, gender-diverse people and people with intersex variations), which actions and activities they deemed most important and the areas of the sector they were most familiar with or interested in influencing. This process was beneficial in capturing those different perspectives and working to account for and align the goals of all stakeholders.

### Strengths and limitations

A key strength of this work was that it was developed in consultation with stakeholders from various academic and professional backgrounds alongside representatives of communities marginalized because of their sex and gender status. These perspectives have been incorporated in the ToC, enabling an expansive view of sex and gender biases in the sectors impacting diverse groups in different ways and conceptualize how we can create change for the benefit of all.

The development of this ToC has some key limitations. First, as this study was conducted from a sector-wide perspective, it is not centred around a specific intervention and does not trace linear pathways of impact, highlight measurable pre-conditions for success or identify parties responsible for certain actions. Rather, it is a broad conceptual model, reflecting the complexity of the problems and potential solutions, and mapping an array of activities, outputs and potential outcomes. Nevertheless, to our knowledge, this is the first such tool for this sector and therefore has potential to be used across the sector to advance sex- and gender-based policy design, evaluation and impact [52]. Second, while this ToC covers a broad range of issues, other connected problems, such as the lack of gender equity in the health and medical research, policy and practice workforce were considered out of scope, though others have clearly linked the two issues [21]. Lastly, we only consulted internal project affiliates for an Australia-based project, and participants were mostly academics, with a small number of end-users working in policy- and community-based organizations. The development of the ToC was based primarily on this consultation, without the benefit of a large literature base, due to the lack of previous research about the efficacy of interventions in this field.

## Conclusions

This paper describes the development of a theory of change (ToC) that maps clear pathways to impact for improving the consideration of sex and gender in health and medical research, policy and practice. This ToC is the first of its kind in the field of health and medical research and provides an important theoretical scaffold for institutions and organizations to consider when considering how to actively enhance sex and gender awareness, inclusivity and informed action to contribute to enhancing population health and economic wellbeing.

### Supplementary Information

Supplementary Material 1.

Supplementary Material 2.

Supplementary Material 3.

Supplementary Material 4.

## Data Availability

The datasets used and analysed under study are available from the corresponding author on reasonable request.
