# Supplementary material for: Theory of change for addressing sex and gender bias, invisibility and exclusion in Australian health and medical research, policy and practice
Source: Health Res Policy Syst. 2024 Jul 15;22:86. doi: 10.1186/s12961-024-01173-z (PMC11251305; doi:10.1186/s12961-024-01173-z)
Supplement: Supplementary file 1 — Supplementary Material 1. [file 12961_2024_1173_MOESM1_ESM.docx]

*Supplementary File 1: SRQR reporting checklist.*

| **Reporting Item** | | **Page number** |
| --- | --- | --- |
| **Title** | Clear title | 0 |
| **Abstract** | Abstract in CONSORT format | 1 |
| **Introduction** | Problem Formulation | 5/6 |
|  | Purpose of research question | 6 |
| **Methods** | Qualitative approach | 7-9 |
|  | Researchers’ characteristics and reflexivity | n/a |
|  | Context | 5 |
|  | Sampling strategy | 8/9 |
|  | Ethical issues | 9 |
|  | Data collection methods | 8-9 |
|  | Data collection instruments | Suppl. 3 |
|  | Unit of study | n/a |
|  | Data processing | 9 |
|  | Data analysis | 9 |
|  | Techniques to enhance trustworthiness | n/a |
| **Results/Findings** | Synthesis and interpretation | n/a |
|  | Links to empirical data | n/a |
| **Discussion** | Integration with prior research/contributions to the field | 18-19 |
|  | Limitations | 19-20 |
| **Other** | Conflict of interest | 0 |
|  | Funding | 0 |
