# Supplementary material for: Theory of change for addressing sex and gender bias, invisibility and exclusion in Australian health and medical research, policy and practice
Source: Health Res Policy Syst. 2024 Jul 15;22:86. doi: 10.1186/s12961-024-01173-z (PMC11251305; doi:10.1186/s12961-024-01173-z)
Supplement: Supplementary file 2 — Supplementary Material 2. [file 12961_2024_1173_MOESM2_ESM.docx]

*Supplementary File 2: Checklist for reporting ToC in Public Health Interventions*

| **Domain/question** | **Page number** |
| --- | --- |
| 1. Is the ToC approach defined? |  |
| 1. Is a definition of ToC given by the authors? | 6 |
| 1. Do the authors explain their reasons for using a ToC approach? | 6 |
| 2. Is the ToC development process described? |  |
| 1. Are the methods used to develop the ToC, such as stakeholder meetings and interviews, document reviews, programme observation, existing conceptual frameworks or published research, described? | 7-9 |
| 1. Where stakeholders are involved, is it clear how many stakeholders participated, what their role is in relation to the intervention, how they were consulted (e.g. number of interviews, focus groups, ToC workshops) and the extent to which the consultations were participatory? | 8/9 |
| 1. Is the method used to compile the data into a ToC described? (including how disagreements between stakeholders were resolved) | n/a |
| 1. Is the extent to which stakeholders were able to validate the resultant ToC and were owners of the final product described? | 11 |
| 3. Is the resultant ToC (or a summary thereof) depicted in a diagrammatic form and does it include? |  |
| 1. The long-term outcome or impact of the intervention | 15-16 |
| 1. The anticipated short- and medium-term outcomes and the process of change | 15 |
| 1. The intervention components which happen at different stages of the pathway | 12-13 |
| 1. The context of the intervention | n/a |
| 1. Assumptions about how change would occur | n/a |
| 1. Additional ToC elements such as indicators, supporting research evidence, beneficiaries, actors in the context, sphere of influence and timelines where relevant. | n/a |
| 4. Is the process of intervention development from the ToC described? |  |
| 1. Are the methods of how interventions were refined from the ToC to something which can be implemented described? (For example, further stakeholder workshops, interviews, systematic literature reviews) | n/a |
| 5. Is the way in which the ToC was used to develop and implement the evaluation described? |  |
| 1. Are evaluation research questions generated from the ToC? | n/a |
| 1. Is the role of ToC in the design, plan or conduct of the evaluation clear? | n/a |
| 1. Does the paper describe the extent to which the key elements described in the ToC were measured in the evaluation (i.e. impact, short- and medium-term outcomes and the process of change, context, assumptions and the intervention)? | n/a |
| 1. Does the paper describe whether and how process indicators were used to improve the quality of the intervention? | n/a |
| 1. Is the role of the ToC in the analysis of the results of the evaluation clear? | n/a |
| 1. Is the role of ToC in the interpretation of the results of the evaluation described? (including the breakdown of programme theory, unanticipated outcomes and causation including the strength and direction of causal relationships) | n/a |
