# Supplementary material for: Theory of change for addressing sex and gender bias, invisibility and exclusion in Australian health and medical research, policy and practice
Source: Health Res Policy Syst. 2024 Jul 15;22:86. doi: 10.1186/s12961-024-01173-z (PMC11251305; doi:10.1186/s12961-024-01173-z)
Supplement: Supplementary file 3 — Supplementary Material 3. [file 12961_2024_1173_MOESM3_ESM.docx]

*Supplementary File 3: Semi-structured Interview Guide.*

**Preamble:** Interviewer

**Introduction:** Interviewer

**Interview Questions**

1. What do you see as the main problems this project is aiming to address? Prompts:
   - In health and medical research practice
   - In the health and medical research sector in Australia
   - In the wider health sector
   - In society
2. What activities need to be done to address these problems? Prompts:
   - By the Sex and Gender Policies in Medical Research Project
   - By the wider sex and gender research field
   - By the sector
3. What are the desired products of these activities?
   - What outputs should be achieved by the project?
   - What other outputs may be produced by these activities across the field?
4. What are the main outcomes that are expected to be achieved by these outputs??
   - From the project
   - From the sector
5. What factors might present a barrier to achieving these outcomes? [Break] What actions might mitigate or address these barriers? [Break] What might facilitate achieving these outcomes?
6. What are the key impacts the project should aim to achieve? Prompts: long-term, wider society
7. Can the project achieve these impacts through the activities, outputs and outcomes discussed? What is needed from outside the project to achieve these impacts? Prompts: Research, Policy, Practice, People
8. Is there anything additional that you think that it is important for us to know, with respect to the objectives of our study?
