# Supplementary material for: Theory of change for addressing sex and gender bias, invisibility and exclusion in Australian health and medical research, policy and practice
Source: Health Res Policy Syst. 2024 Jul 15;22:86. doi: 10.1186/s12961-024-01173-z (PMC11251305; doi:10.1186/s12961-024-01173-z)
Supplement: Supplementary file 4 — Supplementary Material 4. [file 12961_2024_1173_MOESM4_ESM.docx]

*Supplementary File 4: Summary of Theory of Change*

| **Problems** |
| --- |
| 1) Lack of awareness of existing sex and gender biases and how those intersect with other biases in health and medical research, policy & practice |
| 2) Inadequate and biased incorporation of sex and gender into health and medical research |
| 3) Sex and gender-based exclusion from meaningful engagement and participation in health and medical research |
| 4) Lack of evidence-based interventions to address sex and gender biases in research, policy, and practice |
| 5) Inequitable health outcomes between different populations |
| **Activities** |
| 1) Sector-wide discussion on conceptions of sex and gender and their relationship to intersectional factors |
| 2) Development and delivery of education and training on sex and gender concepts, their relevance and application to health research and translation |
| 3) Advocacy and awareness building around current issues and solutions |
| 4) Mapping current knowledge, policy and practice in relation to sex and gender |
| 5) Diverse community and stakeholder involvement and engagement in evidence generation, translation, implementation and evaluation |
| 6) Developing diverse and multidisciplinary networks and communities of practice |
| 7) Policy development and implementation throughout the health and medical research sector |
| 8) Production or implementation of standards for consideration of sex and gender in research design |
| 9) Collection of accurate and inclusive sex and gender related and disaggregated data. |
| 10) Production of standards for consideration of sex and gender in design and regulation of medical products |
| 11) Translation of evidence into clinical guidelines and health policy that explicitly considers different populations |
| 12) Monitoring, evaluation, governance and regulation of health and medical research and translation |
| **Outputs** |
| 1) Shared language for discussing sex, gender and intersectional factors |
| 2) Resources, training, curricula and advocacy material |
| 3) Educated, skilled and aware health research, delivery, policy and governance workforce |
| 4) Baseline reporting on current incorporation of sex and gender in health and medical research, policy and practice |
| 5) Central hub for networking, engagement and resources |
| 6) New or updated policies and guidelines for sex and gender incorporation into health research and practice across the sector |
| 7) Comprehensive and inclusive data and evidence on sex, gender and health |
| 8) S&G informed clinical guidelines, standards, regulations, public policies and strategies |
| 9) Reporting on changes in research practice |
| 10) Reporting on health indicators across sex and gender domains |
| 11) Established feedback mechanisms for continuous monitoring, evaluation and improvement of health research, policy and practice |
| **Outcomes** |
| 1) Improved integration of sex and gender in research design |
| 2) Meaningful, accurate and inclusive data collection and reporting |
| 3) Greater inclusion and participation in health and medical research |
| 4) Recognition and application of sex and gender as nuanced, evolving concepts that intersect with other factors that impact health |
| 5) Multidisciplinary sex and gender networks and communities of practice |
| 6) Improved skills, knowledge and understanding of importance of sex and gender in health/medical research, policy, and practice |
| 7) Increased awareness and use of best practice standards and guidelines |
| 8) Presence, implementation and monitoring of sex and gender research policies in the sector |
| 9) Efficient, inclusive and fit for purpose health interventions and services |
| 10) Health sector held accountable for ongoing action to address gender disparities in health outcomes |
| **Impacts** |
| 1) Better quality, nuanced, and new health data and information |
| 2) Safe, meaningful, and representative participation and experience of diverse groups in health research, policy, practice and care |
| 3) Health and medical sector-wide commitment to reform towards fairer, more inclusive and representative policy and practice |
| 4) Better and more equitable health and economic outcomes |
